# Testing models for the leaf economics spectrum with leaf and whole-plant traits in *Arabidopsis thaliana*

**DOI:** 10.1093/aobpla/plv049

**Published:** 2015-05-08

**Authors:** Benjamin Blonder, François Vasseur, Cyrille Violle, Bill Shipley, Brian J. Enquist, Denis Vile

**Affiliations:** 1 Environmental Change Institute, University of Oxford, South Parks Road, Oxford OX1 3QY, UK; 2 Laboratoire d'Ecophysiologie des Plantes sous Stress Environnementaux (LEPSE), INRA, Montpellier SupAgro, F-34060 Montpellier, France; 3 Centre d'Ecologie Fonctionnelle et Evolutive, CNRS, UMR5175, F-34000 Montpellier, France; 4 Département de Biologie, Université de Sherbrooke, Sherbrooke (Québec), Canada J1K 2R1; 5 Department of Ecology and Evolutionary Biology, University of Arizona, PO Box 210088, 1041 E Lowell St., Tucson, AZ 85721, USA; 6 The Santa Fe Institute, 1399 Hyde Park Road, Santa Fe, NM 87501, USA

**Keywords:** *Arabidopsis*, cytoplasm–cell wall partitioning, genotype, leaf economics spectrum, structural equation modelling, vein density, venation network

## Abstract

The leaf economics spectrum (LES) describes strong relationships between multiple functional leaf traits that determine resource fluxes in vascular plants. Five models have been proposed to explain these patterns: two based on patterns of structural allocation, two on venation networks and one on resource allocation to cell walls and cell contents. Here we test these models using data for leaf and whole-plant functional traits. We use structural equation modelling applied to multiple ecotypes, recombinant inbred lines, near isogenic lines and vascular patterning mutants of *Arabidopsis* *thaliana* that express LES trait variation. We show that a wide variation in multiple functional traits recapitulates the LES at the whole-plant scale. The Wright *et al.* (2004) model and the Blonder *et al.* (2013) venation network model cannot be rejected by data, while two simple models and the Shipley *et al*. (2006) allocation model are rejected. Venation networks remain a key hypothesis for the origin of the LES, but simpler explanations also cannot be ruled out.

## Abstract (precis)

The LES is a global set of patterns that describes correlations between functional traits (e.g. carbon assimilation rate, lifespan) that determine terrestrial resource fluxes. Several classes of models have been proposed to explain the LES, but none have been extensively tested against each other with fine-scale data. Here we assemble a large set of genotypes of *Arabidopsis thaliana* that show variation in leaf functional traits. We show that no extant theory for the LES is supported, but that some leaf venation networks likely play a role in driving the pattern. Our study provides a roadmap for the measurements that could generate further insight into this fundamental ecological pattern.

## Introduction

The leaf economics spectrum (LES) describes correlations between multiple leaf traits, including carbon assimilation rate (*A*_m_), leaf lifespan (LL), leaf mass-per-area (LMA) and nitrogen content (*N*_m_) (Wright *et al*. 2004). These patterns are found globally and across all vascular plant taxa (Reich *et al*. 1997; Wright *et al*. 2005). The trait correlations that define the LES describes a gradient of leaf and plant strategies between ‘live fast, die young’ and ‘live slow, die old’ (Wright *et al*. 2004; Reich 2014). The importance of the LES is clear: it describes an observed variation in plant strategies and highlights strong limitations on terrestrial nutrient fluxes. Moreover, the LES is found at multiple scales including between-species (Wright *et al*. 2004), within-species (Edwards 2006; Dunbar-Co *et al*. 2009; Vasseur *et al*. 2012; Niinemets 2015) and even within-individual plants (Blonder *et al*. 2013).

Recently, several physiological models for the mechanistic origin of the LES have been proposed. Each makes different predictions for the causal relationships and thus correlations between functional traits. However, these models have not been extensively tested against empirical data at the intraspecific scale, which would provide a test of underlying assumptions about physiological mechanisms. Additionally, these models have not been assessed in a genetic context, which can provide insights into the evolutionary mechanisms and genes [e.g. natural selection or genetic constraints (Donovan *et al*. 2011)] that generate the LES. We describe these models below.

### Wright *et al*. (2004) model

In their original paper on the LES, Wright *et al*. (2004) proposed that patterns of trait covariation were due to several independent drivers. Following the interpretation of Shipley *et al*. (2006), high per-mass photosynthetic rates should be associated with large nitrogen investment or low carbon investment due to allocation trade-offs, resulting in *N*_m_ causing *A*_m_ as well as an undetermined covariance between *N*_m_ and LMA. High photosynthetic rates may also be associated with low LL, because at the whole-plant scale, non-optimal resource use could arise if high-performance leaves lived long enough to experience self-shading from canopy growth: thus high *A*_m_ should cause low LL (Ackerly and Bazzaz 1995). Additionally, long LL requires tougher construction that is associated with high LMA, resulting in LMA causing LL. This formulation of the Wright *et al*. model was rejected by Shipley *et al*. (2006), but it has not been tested at the intraspecific scale.

### Meziane and Shipley (2001) model

An earlier model of Meziane and Shipley (2001) used similar logic to derive a slightly different set of predictions. Here, leaf mass is assumed to be primarily allocated to the cell wall, with a smaller allocation to the cytoplasm. Because most nitrogen is in the cytoplasm, higher LMA should displace cytoplasm volume and thus cause lower *N*_m_. Additionally, higher LMA should be associated with thicker leaves and thus lower per-mass photosynthesis, resulting in LMA causing *A*_m_. As in the previous model, nitrogen is assumed to limit photosynthesis, such that *N*_m_ causes *A*_m_. Leaf lifespan can be added to the model in the same way as in the Wright *et al*. (2004) model.

### Shipley *et al*. (2006) model

Shipley *et al*. (2006) applied exploratory methods of causal analysis to the original data of Wright *et al*. (2004) and found that the patterns of trait covariation could only be generated by an unmeasured (‘latent’) variable. They subsequently showed (their Fig. 3) that the patterns of covariation in the full LES data were consistent with a model in which each of *A*_m_, LL, LMA and *N*_m_ was caused by this single unmeasured variable, plus a direct effect from *A*_m_ to LL, as implied by Kikuzawa's (1995) carbon gain optimization theory. Shipley *et al*. (2006) proposed a biological hypothesis that identified this unmeasured variable as the ratio of the volume of the leaf occupied by cytoplasm, *V*_c_, to the volume occupied by cell walls, *V*_w_. Metabolic activity (photosynthesis, respiration) and nitrogen (a key element in these metabolic activities) should scale with the volume of cytoplasm because these processes primarily occur in the cytoplasm. Leaf dry mass should scale with the volume occupied by cell walls, because dry mass occurs mostly in cell walls. Therefore, the mass-based metabolic processes involved in the LES should scale positively with the ratio of *V*_c_ to *V*_w_ (i.e. more metabolic activity per dry mass) while LMA and leaf construction costs (and thus leaf lifetime) should scale negatively with this ratio. While *V*_c_/*V*_w_ is a difficult trait to measure, it should be negatively correlated with easily measured traits such as the ratio of leaf water to leaf dry mass or leaf dry matter content (LDMC), the ratio of leaf dry mass to fresh mass (dimensionless) (Shipley *et al*. 2006). Thus, the cell wall/cytoplasm allocation model predicts that higher values of a latent variable should be associated with lower LDMC, higher *A*_m_, lower LL, lower LMA and higher *N*_m_, in addition to the negative relationship between *A*_m_ and LL. Using 80 species for which LDMC and LES trait data were available, Shipley *et al*. (2006) found preliminary support for this latent variable model.

### Blonder *et al*. (2011) model

Leaf functioning is closely linked to leaf hydraulics and the venation network (Brodribb *et al*. 2010; Sack and Scoffoni 2013). Leaf veins provide multiple functions (e.g. water transport, carbon transport, mechanical support and defense against herbivory). Blonder *et al*. (2011) emphasized a constrained spectrum of leaf trait combinations that are ultimately linked to the geometry of the leaf venation network. In their model, a key venation trait is vein density (VD; mm mm^−2^); i.e. the total length of veins per unit area of the leaf lamina. According to the Blonder *et al*. (2011) model, higher VD increases water transport capacity (i.e. transpiration rate) (Brodribb *et al*. 2007), leading to increased maximum stomatal conductance and increased maximum rate of carbon assimilation (*A*_m_); such high photosynthetic rates then may require higher *N*_m_. Other assumptions about optimizing water flow imply that higher VD would then also decrease leaf thickness (Noblin *et al*. 2008; Brodribb *et al*. 2013). Lower leaf thickness can then lead to lower LMA and lower damage resistance (i.e. lower LL), assuming fixed tissue density. Thus, the original venation model predicts that higher VD should be associated with higher *A*_m_, lower LL, lower LMA and higher *N*_m_. While empirical studies to test this model have been limited, these predictions were generally supported by empirical measurements across 25 species (Blonder *et al*. 2011).

### Blonder *et al*. (2013) model

A recent elaboration of the Blonder *et al*. (2011) model further detailed additional couplings between VD and other leaf traits. Variation in leaf area and thickness, independent of VD, can modulate the LES correlations and, hence, the role of venation networks in the LES (Blonder *et al*. 2013). Depending on the intraspecific scaling relationships between these variables, the sign of other LES correlations can even change. This work leads us to hypothesize that the hydraulic path length (the characteristic distance for water transport from minor veins to stomata, dependent on both VD and mesophyll thickness) may play a more primary role than VD in shaping the LES because it more closely reflects water transport processes within the leaf (Brodribb *et al*. 2007; Boyce *et al*. 2009). Because the hydraulic path length's underlying two variables are relatively difficult to measure simultaneously, this model can be recast in structural equation modelling terms via two variables: an unmeasured (‘latent’) variable and VD. A revised venation-associated model thus predicts that higher values of a latent variable should cause higher VD, higher *A*_m_, lower LL, lower LMA and higher *N*_m_.

Here we test each of these models for the LES by focusing on how well the physiological trade-offs predicted by each model apply across genotypes within the model species *Arabidopsis thaliana*. Our study uses phenotypic variation in *A. thaliana*, using multiple recombinant inbred lines (RILs), several wild-type accessions that span broad climatic gradients, as well as mutants at specific loci relevant to the LES. Previous studies have shown that a large fraction of the global variation in LES traits is expressed across these RILs (Vasseur *et al*. 2012). The set of mutants was chosen to evaluate the impact on the LES of a loss of function in the molecular pathway controlling a physiological process known, or hypothesized, to be associated with LES variation. Our experimental approach allows us to utilize the genomic resources available for this model species in order to assess the genotype–phenotype implications of each model as well as their predictions.

We test these models using two approaches. First, each of these models predicts specific (and differing) causal structures that are reflected in observed patterns of correlation and partial correlation between variables. We use structural equation modelling to determine whether each model could be rejected based on its causal structure and the observed data. Second, we can build on previous genetic studies to directly manipulate the genes hypothesized to underlie variation in the LES, and so determine whether such manipulations are consistent with hypotheses for leaf physiology. Because the LES implies strong limitations for leaf-level nutrient fluxes, it is also associated with important variations on whole-plant growth strategy (Freschet *et al*. 2010; Reich 2014). Recently, Vasseur *et al*. (2012) identified a small set of pleiotropic genes at two *A. thaliana* loci (*EDI* and *FLG*) that are involved in the variation of multiple functional traits related to the economy of carbon and nitrogen at the leaf and whole-plant level. These findings provide a set of mechanistic linkages between genes, physiology and trait correlations.

We apply both of the above approaches to assess the origin of the LES using multiple genotypes of the model plant *A. thaliana*. We assembled a dataset of 31 genotypes, including (i) five ecotypes with geographic ranges in the Northern hemisphere, (ii) 16 RILs generated by crossing two ecotypes from contrasting environments, (iii) five near isogenic lines (NILs) varying from wild-type only at loci previously identified to control LES variation and (iv) five knockout mutants, of which three have loss of function in candidate genes found within the above loci and two have reduced auxin sensitivity, causing vascular patterning defects. These genotypes have been extensively studied via molecular and phenotyping approaches (El-Assal *et al*. 2001; Doyle *et al*. 2005; Keurentjes *et al*. 2007; Fu *et al*. 2009; Vasseur *et al*. 2012) and now provide a wide range of phenotypes with known genetic causes. We grew replicates of each genotype under controlled environmental conditions and used a high-throughput phenotyping system (Granier *et al*. 2006) to study variations in functional traits (*A*_m_, measured at the rosette level; LL, measured at the rosette level by flowering time; LMA, measured at the rosette level; *N*_m_; measured at the rosette level) as well as two possible predictor traits (VD, measured at the leaf level; and LDMC, measured at the rosette level). This study of functional covariation effectively extends the LES to the whole-plant scale (Vasseur *et al*. 2012).

## Methods

### Plant material

We selected five *A. thaliana* ecotypes originating in multiple environments (Col-0 and Col-4, originating in Germany; Cvi-0, originating in the Cape Verde Islands; L*er*-0, L*er*-2, from irradiated seeds originating in Poland). We also selected a subset of 16 contrasting RILs from a L*er* × Cvi population (Alonso-Blanco *et al*. 1998). The phenotypes of these RILs cover the range of leaf trait values previously observed for a larger set of RILs from the same population (Vasseur *et al*. 2012). We also selected five NILs developed by introgressing Cvi regions into L*er* background (Keurentjes *et al*. 2007) in order to assess the *EDI* and *FLG* loci, which are known to have multiple pleiotropic effects on the LES (Vasseur *et al*. 2012). Near isogenic lines 1–2.13, 1–2.5 and 1–3 carry introgressions of chromosome 1 associated with the *EDI* locus. Near isogenic lines 5–7 and 5–8 carry introgressions of chromosome 5 associated with the *FLG* locus. We also selected three knockout mutants for two candidates genes [*CRY2*, encoding a blue-light receptor involved in circadian regulation and reproductive transition (El-Assal *et al*. 2001), and *HUA2*, a repressor of floral transition (Doyle *et al*. 2005) known to be implicated in the *EDI* and *FLG* loci (Doyle *et al*. 2005)]. Mutants *cry2-1* (Col-4 background) and *fha-1* (L*er*-0 background—http://arabidopsis.info/StockInfo?NASC_id=108) describe loss of function in *CRY2*, while mutant *hua2-4* describe loss of function in *HUA2* (Col-0 background). Finally, we also selected two knockout mutants for the *AXR1* gene that confers resistance to auxin, a hormone involved in leaf vascular patterning (Alonso-Peral *et al*. 2006; Scarpella *et al*. 2010). Mutants *axr1-3* and *axr-12* (both Col-0 background—http://arabidopsis.info/StockInfo?NASC_id=3075, http://arabidopsis.info/StockInfo?NASC_id=3076) are associated with incomplete leaf vascular development and lower VD.

### Growth conditions

**Figure 1.**
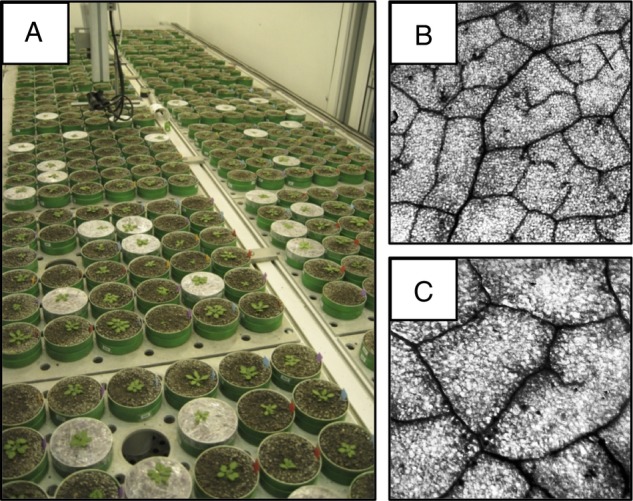
(A) We use the PHENOPSIS automated phenotyping platform to grow many plants under tightly controlled conditions. Within these genotypes, venation network geometry is highly variable. Shown are two RILs from the Ler × Cvi cross: (B) with VD = 3.2 mm^−1^; (C) with VD = 1.9 mm^−1^. Full width for both images, 3 mm.

We used the PHENOPSIS automated growth chamber facility (Granier *et al*. 2006; Fabre *et al*. 2011) to grow and phenotype the plants (Fig. 1A). This facility can maintain constant environmental conditions and take zenithal photographs of the plants. Briefly, plants were watered daily, with conditions standardized at 20 °C temperature and a 12 h light (200 µmol m^−2^ s^−1^)/12 h dark illumination cycle. For this study, we grew a total of 199 plants (*n* = 7.0 ± 2.2 s.d. per mutant, 6.4 ± 0.5 per NIL, 5.4 ± 0.7 per RIL and 9.0 ± 2.9 per ecotype). A full description of growth conditions can be found in Appendix 1 of Vasseur *et al*. (2012).

### Trait measurements

All traits were collected at first flowering after removing flowering stems from the rosette. Leaf metabolism and hydraulic properties change dramatically during leaf development, leading to a transition from sink to source organ that varies between genotypes and among environmental conditions (Pantin *et al*. 2011). When possible, we measured leaf traits; otherwise, we measured whole-plant traits. Leaf traits were measured on fully expanded leaves to avoid trait variation due to source/sink differences among genotypes with different developmental trajectories. However, whole-plant traits may be influenced by source/sink differences among genotypes with different developmental trajectories (Pantin *et al*. 2011).

To measure whole-plant photosynthesis, we used a whole-plant chamber designed for *A. thaliana* and connected to an infrared gas analyzer (CIRAS 2; PP Systems, Amesbury, MA, USA). Before making measurements, we sealed the surface of the soil with plastic film to eliminate carbon fluxes from soil respiration. *A*_m_ (nmol g^−1^ s^−1^) was determined at the time of flowering as the whole-plant rate divided by the total mass of leaf blades, under ambient light and CO_2_ levels. Our whole-plant metric of *A*_m_ has several benefits. First, this metric is tractable to measure given the very small area of some genotypes’ leaves. Second, this metric is taken at a standardized developmental time-point for the whole plant, and leaf traits are often strongly correlated with whole-plant traits (Freschet *et al*. 2010), representing broader trade-offs at the whole-plant scale (Grime 1977).

Time to flowering is a key trait in annual species because longer flowering time is associated with bigger, thicker and older leaves. Senescence generally occurs shortly after flowering, so there are strong correlations between LL, whole-plant lifespan and age at flowering [see, for instance, the Supplemental Material of Vasseur *et al*. (2012)]. To estimate the whole-plant life-history cost of the variations in LES strategies, we measured LL (in day) using flowering time as a genetically determined proxy (Salomé *et al*. 2011). We also calculate LMA and other traits at the whole-plant level. We first harvested and weighed each rosette at flowering to get total leaf (blades + petioles) fresh mass. We then wrapped the rosette in moist paper and kept it at 4 °C overnight to fully rehydrate the leaves. The oldest non-senescing and fully expanded leaf was then stored in a vial at −80 °C to be later measured for VD. For all remaining leaves, the total leaf area was obtained by separating the blades from the petioles of all leaves, and digitally scanning each leaf. Blades and petioles were then dried at 65 °C for 72 h and weighed separately to determine total blade and total petiole dry mass. Leaf dry matter content (mg mg^−1^) was calculated as total leaf dry mass divided by total leaf fresh mass (blades + petioles). Leaf mass-per-area (g m^−2^) was then determined as the total blade dry mass divided by the total blade area. The range of LMA values we observed for individuals in this herbaceous species (14–69 g m^−2^) may seem high. However, our range of LMA values is similar to the range of values previously published for this species [e.g. 3.2–8.8 g m^−2^ (Bonser *et al*. 2010); 13–32 g m^−2^ (El-Lithy *et al*. 2010); 14–67 g m^−2^ (Des Marais *et al*. 2012; (Vasseur *et al*. 2012)]. The deviation in our range of LMA values with other studies (e.g. Li *et al*. 1998) is likely because of the differences in the growth conditions and developmental stage in which LMA was measured. Vein density was measured at leaf scale by a chemical clearing process and subsequent digital imaging and hand-tracing (Fig. 1B and C). Leaves were defrosted and cleared in a solution of 0.5 % safranin in ethanol for 7 days, then rinsed in a series of ethanol, 1 : 1 ethanol : toluene, and toluene before slide-mounting in the toluene-based Permount resin. After curing for 3 days, slides were back-illuminated and imaged using a dissecting microscope (SZX12, Olympus) and digital camera (T2i, Canon). Final image resolution was 195 pixels per millimetre. A contrast-limited adaptive histogram equalization was applied to the red channel of each image to enhance the image quality. Using an image-editing programme (GIMP, GNU), we hand-traced all veins within a section of each leaf. We then calculated leaf section area, and leaf section vein length using a custom programme (MATLAB, MathWorks). Vein density was then calculated as leaf section vein length divided by leaf section area. We hand-traced only a section of each leaf because some leaves did not completely clear or were mechanically torn during the slide-mounting process. We therefore identified one or more polygonal sections of each leaf that was fully cleared, as determined by high vein-lamina image contrast and/or clear evidence of minor veins or freely ending veinlets. We traced a mean sectional area of 52 mm^2^ (±30 s.d.) per sample, consistent with community guidelines (Pérez-Harguindeguy *et al*. 2013).

We obtained whole-plant estimates of *N*_m_ by measuring at least one individual for every genotype. *N*_m_ (g g^−1^) was determined on all dried leaves pooled together (without petioles) by mass spectrometry (EA20000, Eurovec; Isoprime, Elementar, Cheadle, UK). For some RILs, genotype-mean *N*_m_ values were obtained from measurements of the same genotypes grown under identical conditions in Experiment 1 of Vasseur *et al.* (2012).

### Statistical analysis

All analyses were conducted in R (http://www.r-project.org). Standardized major axis regressions were made with the ‘smatr’ package. Power analyses were made with the ‘pwr’ package. Structural equation models were tested with the ‘lavaan’ package.

All structural equation models were built using genotype-mean values. Mean values were then log_10_-transformed to match the normality assumptions of structural equation modelling. We fit each candidate model using a Wishart likelihood (Wishart 1928), which can compensate for any remaining non-normality in the data. We report lack-of-fit *P*-values for each model that correspond to a maximum likelihood *χ*^2^ statistic.

### Data availability

Datasheets and R code to replicate all analyses are available in **Supporting Information**. Pictures of leaf blades and petioles as well as raw biomass measurements are available at http://bioweb.supagro.inra.fr/phenopsis/ under experiment C3M13B (Fabre *et al*. 2011). Images of chemically cleared leaves (greyscale) and hand-traced subregions (annotated with yellow for fully cleared regions; with red for veins) are available at http://clearedleavesdb.org under the collection name ‘*Arabidopsis thaliana* genotypes’.

## Results

**Figure 2.**
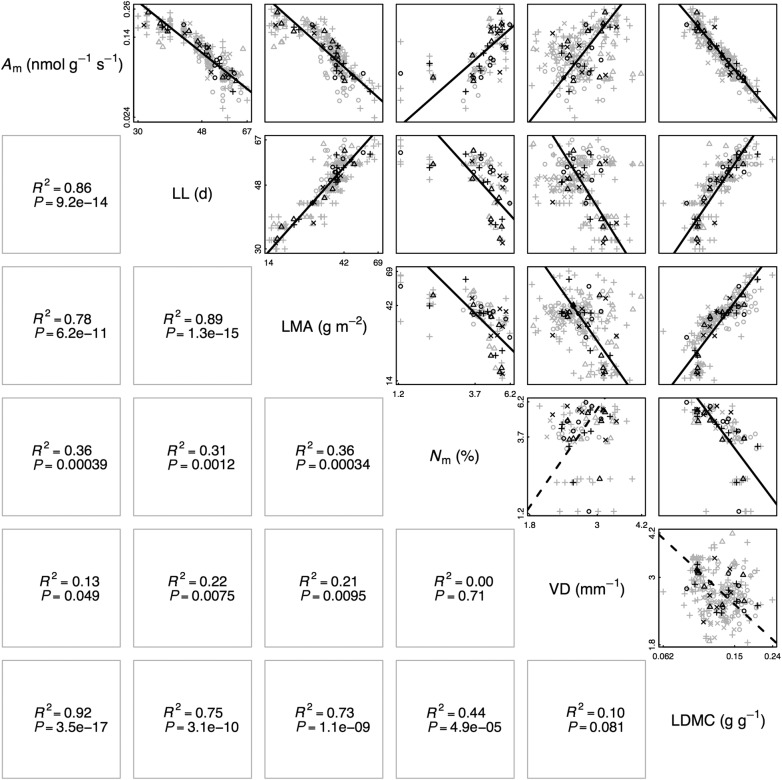
Pairwise correlations between LES traits, VD and LDMC for multiple *A. thaliana* genotypes. Grey points indicate individual leaves; black points, genotype means. Symbols indicate genotype category: circles, mutants; triangles, NILs; pluses, RILs, crosses, ecotypes. Pairwise relationships between traits are shown, with lines indicating standard major axis regressions on genotype-mean log_10_-transformed data. Solid lines, *P* < 0.05; dashed lines, not significant. Regression statistics are shown in the lower panel for each pairwise relationship.

We first assessed correlations between LES traits. Consistent with a previous demonstration (Vasseur *et al*. 2012), LES correlations between *A*_m_, LL, LMA and *N*_m_ were found across genotypes (Fig. 2). All pairwise correlations between these genotype-mean values were significant (standard major axis regressions, all *P* < 0.05, *R*^2^ > 0.31), with signs the same as those described by the interspecific LES (Wright *et al*. 2004).

Leaf dry matter content and VD, the core traits of several of the models tested here, both showed wide ranges of variation. Vein density varied between 1.8 and 4.2 mm^−1^ and LDMC varied between 6.2 and 23.9 %. Both variables were significantly correlated with all LES traits (except for a non-significant correlation between VD and *N*_m_). Correlations between LES traits with LDMC were generally higher than with VD.

We next compared causal models for these LES correlations. The conceptual model of Wright *et al*. (2004) could not be rejected (*χ*^2^ = 1.28, df = 1, *P* = 0.26) and had coefficients with signs consistent with its original formulation. However, the conceptual model of Meziane and Shipley (2001) was strongly rejected (*χ*^2^ = 14.7, df = 2, *P* < 10^−3^). We then tested the Shipley *et al*. model that includes LDMC as an observed proxy of the latent variable *V*_c_/*V*_w_. The overall model was rejected (*χ*^2^ = 30.1, df = 4, *P* < 10^−5^) (Fig. 3A). We also found that the Blonder *et al.* (2013) model converged on coefficients with signs consistent with its original formulation (Fig. 3B), but the overall model was rejected (*χ*^2^ = 133.3, df = 6, *P* < 10^−15^). However, the revised venation-associated model was not rejected by data (*χ*^2^ = 7.8, df = 5, *P* = 0.16) and also converged on coefficients with signs consistent with predictions (Fig. 3C).

**Figure 3.**
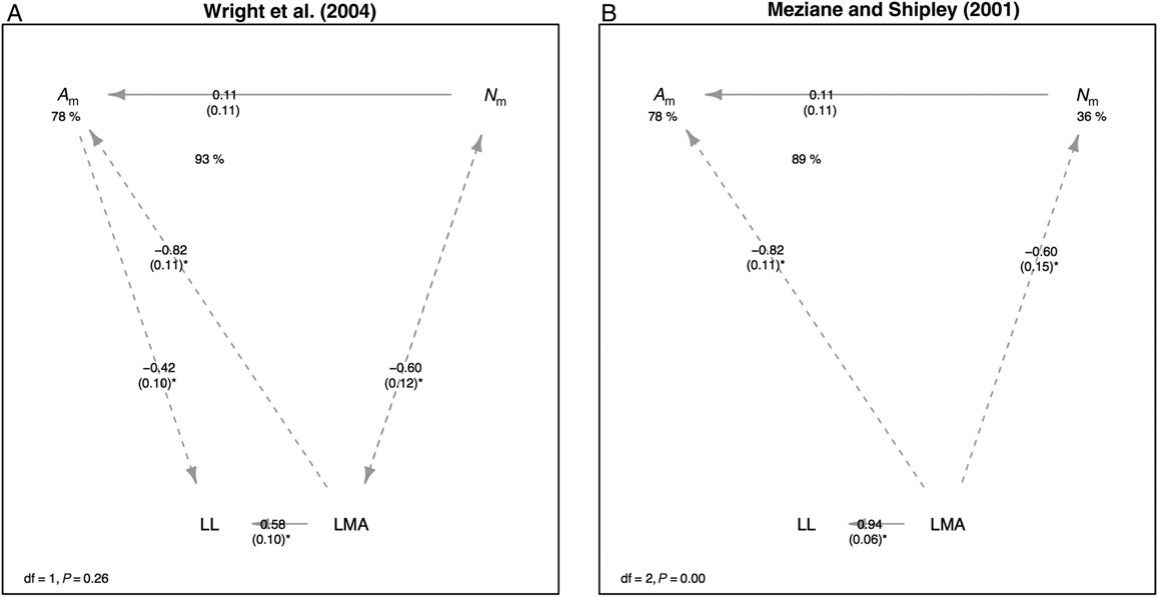
Empirical evaluation of five possible models for the LES: (A) the model of Wright *et al*. (2004), (B) the model of Meziane and Shipley (2001), (C) the model of Shipley *et al*. (2006), (D) the venation model of Blonder *et al*. (2011) and (E) the revised venation-associated model of Blonder *et al*. (2013). Labels on arrows indicate standardized path coefficients, with standard errors in parentheses and an asterisk if significant at the *α* < 0.05 level. Paths are drawn with solid line if positive and dashed if negative. Values under dependent variables indicate *R*^2^ values. The overall model degrees of freedom and lack-of-fit *P*-value are shown in the legend; larger *P*-values indicate that the model is less likely to be rejected by observed data. Models are calculated using log_10_-transformed genotype-mean trait data.

**Figure 3.**
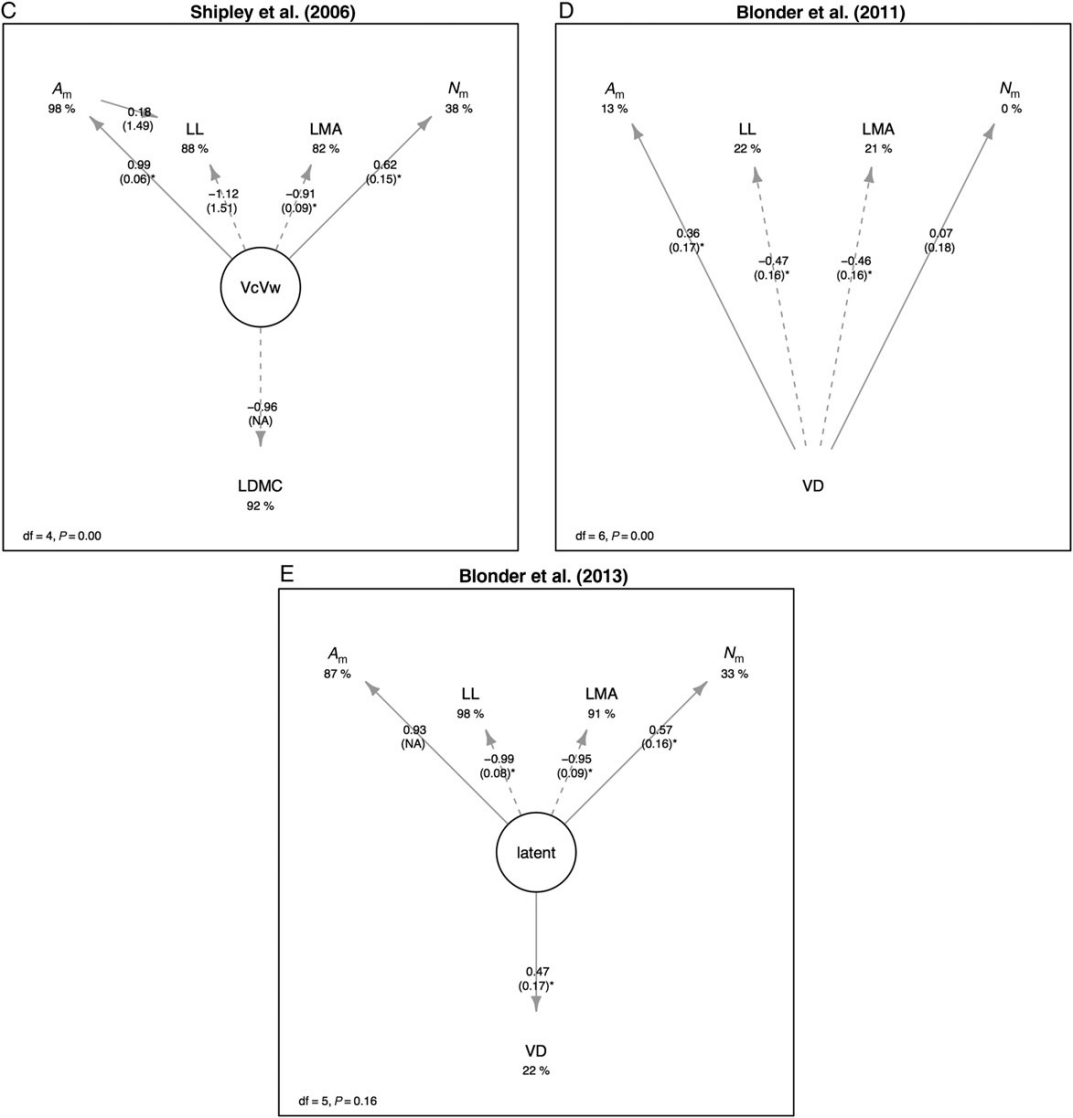
Continued.

We then compared relative support for the two models that could not be rejected by using AIC-based model selection. We found that the revised venation-associated model (Blonder *et al*. 2013) had less information loss (ΔAIC = 90) than the Wright *et al.* (2004) model. This result indicates that both models are plausible explanations of the data, but the revised venation-associated model is far more likely, even after correcting for differences in the number of free parameters in each model.

**Figure 4.**
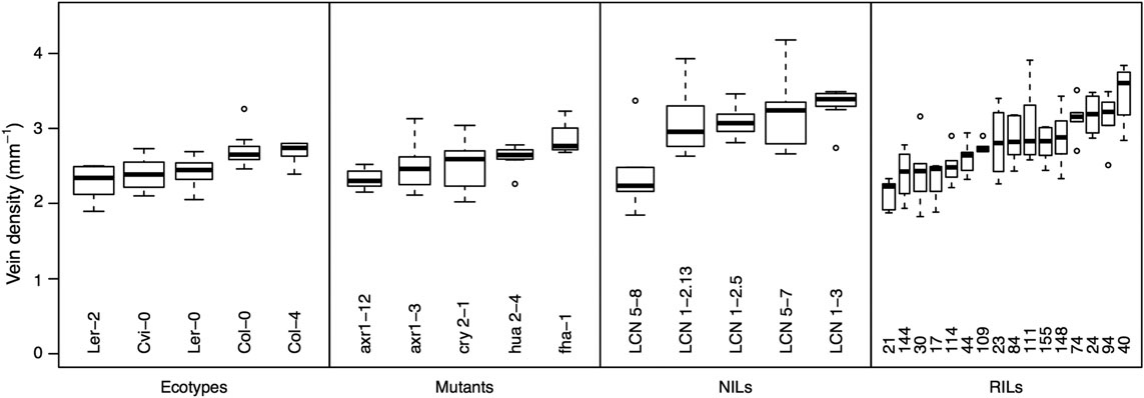
Vein density varies threefold between *A. thaliana* genotypes. This variation is found between ecotypes, mutants generated to target loci associated with the LES or vascular development, NILs or RILs generated from crosses between ecotypes.

We also determined whether the genotypes known to show an LES trait variation also showed a variation in VD, a necessary condition for a venation network hypothesis to be supported. We found a significant variation in VD among ecotypes (ANOVA, *F*_4,41_ = 5.7, *P* = 0.0009) and among RILs (ANOVA, *F*_15,70_ = 7.0, *P* < 10^−8^) (Fig. 4). Using the NILs, we found that each line tied to the *EDI* locus (LCN 1–2.13, LCN 1–2.5 and LCN 1–3) had significantly different VD from the parent L*er*-2 ecotype (*t*-tests, all *P* < 0.01). For the FLG locus, the LCN 5–7 line had significantly different VD than the parent (*P* = 0.003), but the LCN 5–8 line did not (*P* = 0.72). Using the mutants, we found that, for the *CRY2* knockout, *fha-1* had different VD values than its L*er*-0 background (*P* = 0.01). In contrast, VD for *cry2-1* was not different from VD in its Col-4 background (*P* = 0.18). For the *HUA2* knockout, VD in *hua2-4* was not different from VD in its Col-0 background (*P* = 0.39). We also tested two *axr1* mutants directly affected in their venation network because of an impairment of auxin signalling. We found that the mutant with the more altered venation pattern, *axr1-12*, had different VD than its Col-0 background (*P* = 0.003) but did not find a difference relative to the mutant with the less altered venation pattern, *axr1-3* (*P* = 0.21).

## Discussion

In the revised venation-associated model, we were not able to directly identify the latent variable controlling correlations between LES traits and VD. We hypothesize that it is linked to leaf hydraulics, based on theory and on the empirical result that the venation pattern-altered mutants also exhibited variations in LES traits. Nevertheless, the weak direct correlation between VD and other LES traits (all *R*^2^ < 0.13) suggests that the key trait is not VD but shows some relationship to it. Indeed, this latent variable may represent a combination of unknown but real physical variables.

Several other variables may also influence leaf economics via modulation of hydraulic conductance and total carbon cost. For example, variation in tissue density (Sack *et al*. 2013), mesophyll thickness (Brodribb *et al*. 2007), variation in vein size (Sack *et al*. 2012, 2013) or stomatal traits (Brodribb *et al*. 2013) may also influence the LES. For example, we note that a positive correlation between VD and LDMC was expected (Blonder *et al*. 2013) but not found here, suggesting that accounting for the variation in vein size would be useful.

We note that a ‘flux network theory’ was also recently proposed as an alternative model for venation models of the LES (Sack *et al*. 2013). In this framework, the LES emerges from interactions between a much wider suite of plant traits associated with resource fluxes and stocks. The validity of the many vs. few trait perspectives remains controversial (Blonder *et al*. 2014). This study was aimed at testing well-known specific few-trait models and was not able to assess this other class of hypotheses.

Our study also provides a roadmap for linking vascular patterning genes to the LES. Building on the work of Vasseur *et al*. (2012), we identified a role for the *EDI* and *FLG* loci, as well as for the *AXR1* and *CRY2* genes and potentially the *HUA2* gene in leaf function and vascular development for *A. thaliana*. Further work may elucidate the molecular or developmental mechanisms coupling these genes to key functional traits, which—with the exception of *AXR1* (Alonso-Peral *et al*. 2006)—remain unknown. Decades of study have yielded many more vascular patterning mutants in *A. thaliana* than were studied here (Pérez-Pérez *et al*. 2002; Scarpella *et al*. 2010). Generally, vascular development follows patterns of auxin concentration and is, therefore, sensitive to the presence of genes that modulate production and inhibition of auxin or sensitivity to it (Donner and Scarpella 2009). Determining whether the genes described above, as well as others at the *EDI*/*FLG* loci, are implicated in these pathways would further assess the hypotheses proposed here. With the progresses made in next-generation sequencing technologies, it is now possible to conduct genome-wide association studies (GWAS) on large panels of natural accessions in model species, as well as in many wild and cultivated species. Testing mechanistic models and causal relationships will be a powerful tool to investigate the mechanistic causes, the genetic determinism and the evolution of the LES.

Some caution should be taken when considering the generality of these results. We compared 31 genotypes of one species under a single set of environmental conditions. The processes generating the interspecific LES may differ from those generating the intraspecific LES. Moreover, a part of the variability in LES traits observed here may be explained by the functional variability associated with changes in flowering time, such as self-shading, reflecting more the whole-plant strategies for the economy of carbon and nutrients. If processes are scale- and species-dependent, then the global convergence in leaf functioning could be a common pattern arising from multiple processes. Many vascular plant species have vastly different life histories than *A. thaliana*, so it is plausible that multiple genetic pathways are responsible for the strong observed convergence in LES with and across taxa.

Data scale issues may also limit the generality of our conclusions. This study used a mixture of leaf-level measurements (VD) and whole-plant measurements (*A*_m_, *N*_m_, LDMC and LMA and LL), which may conflate the LES (Wright *et al*. 2004) with the whole-plant economics spectrum (Reich 2014). This could reduce or bias correlations: for example, our measurements of *A*_m_ and LMA include leaves of different ages, while VD is measured for a single leaf for each individual. There may also be a biased relationship between average LL and flowering time, because of the non-linear increase in leaf number with plant size. However, our whole-plant metrics are strongly correlated with leaf-level metrics in this system (Vasseur *et al*. 2012) and the small size and short lifespan of *A. thaliana* (i.e. no allocation to woody tissue) suggest that leaf and plant ecological strategies are probably tightly coordinated in this species. All models tested here were built to explain variations in the strategies of resource acquisition and use at the leaf level to, in turn, explain variations in whole-plant growth strategies and adaptation to variable environments. Our results indicate an important contribution of leaf-level functional variability to variations in plant economics. On the other hand, our study also pinpoints the role of an unknown variable on trait variation, which suggests that plant architecture and leaf geometry may also be part of strategies to optimize biomass allocation and nutrient-use efficiency.

Some of our null results (e.g. no significant difference in VD between a vascular patterning mutant and wild-type) may also be due to low statistical power. For an effect size of 0.5 (reasonable given the difference in means and error variances observed in these results) and a power of 0.9 (i.e. a 10 % chance of incorrectly deciding there is no difference in VD), a sample size of ∼70 would be required for these two-sided *t*-tests. Sample sizes for ecotypes, mutants and NILs were never larger than 12. However, the significant differences we did find are likely to represent true genetic effects on VD.

Lastly, these data and our analyses do indicate that the trait correlations seen in the LES are not just a statistical artefact of data normalization (Lloyd *et al*. 2013; Osnas *et al*. 2013). Correlations not reflective of physiological mechanism might be found when examining pairs of variables where one is a ratio of another and scale isometrically with each other (e.g. *A*_m_ negatively correlated with LMA with a slope of −1.0). However, we found evidence that these correlations, as well as all others, are not driven simply by these statistical effects but rather by variations in a latent variable representing one or more physical quantities. Thus, our study supports the idea that LES trait correlations have a physiological basis and contribute strongly to variations in the whole-plant economic strategies.

## Conclusions

Our data provide a starting point for assessing the functional basis of the LES in *A. thaliana*. Using structural equation modelling, we showed that the original conceptual model of Wright *et al*. (2004) could not be rejected by data. However, a revised venation-associated model (Blonder *et al*. 2013) is also consistent with the observed data for multiple genotypes whose phenotypes recapitulate the interspecific LES (Vasseur *et al*. 2012). We suggest that the latter model is more plausible, because (i) the Wright *et al*. model was rejected at the interspecific scale in a previous study (Shipley *et al*. 2006) and (ii) model-selection techniques indicated more relative support for the revised venation-associated model. Nevertheless, we emphasize that both models remain viable explanations for the LES given the extant data in *A. thaliana*.

## Sources of Funding

B.B. was supported by an NSF pre-doctoral fellowship. F.V. was funded by a CIFRE grant (ANRT, French Ministry of Research) supported by BAYER Crop Science (contract 0398/2009 - 09 42 008). C.V. was supported by a Marie Curie International Outgoing Fellowship within the 7th European Community Framework Programme (DiversiTraits project, no. 221060) and by the European Research Council (ERC-2014-StG-CONSTRAINTS grant). B.S. was funded by the Natural Sciences and Engineering Research Council of Canada. B.J.E. was supported by a NSF Advancing Theory in Biology award. D.V. was supported by EIT Climate-KIC program AgWaterBreed.

## Contributions by the Authors

B.B., C.V. and B.J.E. conceived the manuscript. B.B., F.V., C.V. and D.V. contributed to laboratory analyses. All authors contributed to statistical analyses and writing the manuscript.

## Conflict of Interest Statement

None declared.

## Supporting Information

The following additional information is available in the online version of this article –

**File S1.** CSV file. Trait measurements for all individual plants.

**File S2.** R code. Scripts to replicate analyses and figures.

Additional Information
