# Supplementary material for: Testing models for the leaf economics spectrum with leaf and whole-plant traits in Arabidopsis thaliana
Source: AoB Plants. 2015 May 8;7:plv049. doi: 10.1093/aobpla/plv049 (PMC4481546; doi:10.1093/aobpla/plv049)
Supplement: Additional Information [file supp_7_plv049_index.html]

Testing models for the leaf economics spectrum with leaf and whole-plant traits in Arabidopsis thaliana — Additional Information 

# Testing models for the leaf economics spectrum with leaf and whole-plant traits in *Arabidopsis thaliana*

## Additional Information

Additional Information

- Additional Information - txt file
- Additional Table - csv file
